# Current Understanding of the Role of PPAR*γ* in Gastrointestinal Cancers

**DOI:** 10.1155/2009/816957

**Published:** 2009-10-26

**Authors:** Bing Zou, Liang Qiao, Benjamin C. Y. Wong

**Affiliations:** ^1^Department of Medicine; Centre Cancer Research, The University of Hong Kong, Pok Fu Lam Road, Hong Kong; ^2^Department of Gastroenterology and Hepatology; Storr Liver Unit, Westmead Millennium Institute, University of Sydney at Westmead Hospital, Westmead, NSW 2145, Australia

## Abstract

Numerous studies have indicated that PPAR*γ* plays multiple roles such as in inflammation, cell cycle control, cell proliferation, apoptosis, and carcinogenesis, thus PPAR*γ* contributes to the homeostasis. Many in vitro studies have showed that ligand-induced activation of PPAR*γ* possess antitumor effect in many cancers including CRC. However, the role of PPAR*γ* in gastrointestinal cancers, especially in colorectal cancer, is rather controversial. Nevertheless, some recent studies with the positive results on the possible application of PPAR*γ* ligands, such as Bezafibrate or Rosiglitazone in gastrointestinal cancers, have suggested a potential usefulness of PPAR*γ* agonists in cancer prevention and therapy. In this review, the authors discuss the recent developments in the role of PPAR*γ* in gastrointestinal cancers.

## 1. An Overview of PPAR Family

Peroxisome proliferator-activated receptor (PPAR) is a member of a family of nuclear hormone receptors that consists of three isoforms: PPAR*α*, PPAR*γ*, and PPAR*δ* (also known as PPAR*β*). Within this family are also retinoid X receptor (RXR), vitamin D receptor, and the thyroid hormone receptor. PPARs act as a ligand-activated transcription factor and are involved in many different biological functions. Extensive study of PPARs was probably sparkled by the identification of PPAR*α* in 1990 [1], which was soon followed by the identification of two other members PPAR*γ* and PPAR*δ* [2, 3]. Each isoform of PPARs is encoded by a separate gene and exhibits different tissue distribution patterns. For example, PPAR*α* is principally expressed in tissues that exhibit a high rate of fatty acid metabolism (e.g., brown adipose tissue, liver, kidney, and heart) and is the primary target for the fibrate class of drugs [4]. PPAR*δ* is ubiquitously expressed in many tissues, and its physiological roles are multiple, including but may not be limited to lipid trafficking [5, 6], blastocyst implantation [7], wound healing [8], and the regulation of fatty acid catabolism and energy homeostasis [9, 10]. PPAR*γ* is richly expressed in adipose tissue, intestinal epithelial cells [11, 12], and macrophages. Low level of PPAR*γ* has also been found in skeletal muscle [13].

Like other nuclear receptors (NRs), all PPARs share a similar modular structure with functionally distinct domains called A/B (ligand-independent activation domain), C (DNA binding domain), D (hinge domain), and E/F (ligand-binding domain, LBD) (Owen et al. [14]). The N-terminal domain A/B has been relatively well conserved through evolution, whereas the C domain is the most conserved of all the functional domains. The less conserved domain D functions as a flexible hinge between the C and E/F domains and contains a sequence recognized by transporting proteins. Some of the amino acids are involved in the activities of nearby domains, leading to the dimerization and recognition of the target DNA sequences (Owen et al. [14]). The largest domain is the LBD located at the C-terminus [15], which is responsible for the binding of a specific ligand to PAR receptors, and subsequent activation of PPAR through binding to peroxisome proliferators response elements (PPREs) on the promoter region of the target genes. Thus, LBD is the major functionally related domain of the PPARs.

PPARs seem to regulate gene transcription by two mechanisms: transactivation and protein-protein interaction with other transcription factors. Transactivation of PPARs is a DNA-dependent mechanism, which involves binding of the PPAR ligands and heterodimerization between PPARs and RXR (Retinoid X receptor) [16]. The heterodimer between PPARs and RXR then binds to PPRE, resulting in stimulation of transcription. In contrast, the protein-protein interaction mechanism involves the activation of target genes through other transcription factors, such as AP1, NF-*κ*B, Smads, STATs, and NFATs. It is suggested that most of inhibitory effects of PPARs are mediated by this mechanism [17, 18].

In this review, we only focus on the controversial role of PPAR*γ* human gastrointestinal cancers.

## 2. PPAR*γ*

In human, the PPAR*γ* gene is located on chromosome 3 at position 3p25.2 [19]. Two isoforms of PPAR*γ* have been identified: PPAR*γ*1 and PPAR*γ*2. These two isoforms only differ in their N termini sequences: PPAR*γ*2 protein contains an additional 30 N-terminal amino acids, but is otherwise identical to PPAR*γ*1. Both PPAR*γ*1 and PPAR*γ*2 N termini function as translational initiators, but the activity of PPAR*γ*2 is higher than that of PPAR*γ*1, suggesting that PPAR*γ*1 and PPAR*γ*2 N termini have distinct activation capacities, and perhaps may have different functions. PPAR*γ*2 is predominantly expressed in adipose tissue, and it has been demonstrated that PPAR*γ*2 N termini is more important in the process of adipocyte differentiation and metabolism [20, 21]. In contrast, PPAR*γ*1 is relatively stable and expressed at very high levels in the gastrointestinal epithelium [12, 22, 23], and low levels were observed in many other tissues [24].

The function of PPAR*γ* relies on its interactions with a coactivator or corepressor. Binding of PPAR*γ* to a coactivator affects the chromatin structure through acetylation of histones, whereas binding of PPAR*γ* to a corepressor alters the chromatin structure through deacetylation of histones. Both coactivators and corepressors are highly versatile and are not specific for particular PPAR subtypes [25]. Binding of PPAR*γ* with coactivators may be either ligand-dependent or ligand-independent. Most coactivators interact with the LBD of NRs utilizing the LXXLL helical motifs in a ligand-dependent manner [26, 27]. In contrast, PPAR*γ* coactivator-1*α* (PGC-1*α*) binds to the hinge domain of PPAR*γ* in a ligand-independent manner [28]. In addition to the ligand-dependent and ligand-independent activation of PPAR*γ*, the activity of this transcription factor may also be modulated by posttranscriptional modification, such as phosphorylation [29–31].

## 3. PPAR*γ* Ligands

Over the past several years, various natural and synthetic PPAR*γ* ligands have been identified, and new ligands are under fast development.

In the broad sense, these ligands include specific PPAR*γ* agonists [32], PPAR*γ* partial agonists [33], and PPAR*α*/*γ* dual agonists [34]. Synthetic PPAR*γ* agonists are able to modulate the adipocyte differentiation, and thus have been used as potential antidiabetic drugs [20, 32, 33]. The most commonly used PPAR*γ* agonists are Thiazolidinediones (TZDs), which include Troglitazone (Rezulin), Pioglitazone (Actos), and Rosiglitazone (Avandia).

TZDs are widely used in animal studies and clinical trials to investigate the role of PPAR*γ*. The roles of PPAR*γ* ligands are multiple. Some TZDs have been licensed for use in patients with Type 2 diabetes mellitus (T2DM) [35], some may benefit cardiovascular parameters, such as lipids, blood pressure, inflammatory biomarkers, endothelial function, and fibrinolytic state [36, 37]. Moreover, they have been successfully used in nondiabetic insulin-resistant conditions such as polycystic ovary syndrome [38, 39]. The synthetic PPAR*γ* ligands, however, are associated with various side effects, such as increased adiposity, edema, hepatotoxicity, and cardiac hypertrophy. Therefore, partial PPAR*γ* ligands with weaker side effects such as LSN862 have been developed [33, 40], and newer PPAR*γ* ligands that do not fall into the category of TZDs are under active development and their biological activities have been tested in various cancer cells. For example, the roles of LY293111 (Eli Lilly), CS-7017 (Sankyo), Spirolaxine (Sigma-Tau), and TZD-18 (Merck) have been investigated in various in vitro systems, and some are under clinical trials [41–45].

In addition to synthetic ligands, some endogenous (or natural) compounds are potent activators for PPAR*γ*. Among the natural PPAR*γ* ligands is cyclopentone 15-deoxy-E12,14-prostaglandin J2 (15d-PGJ2). This agent is probably the most potent endogenous PPAR*γ* ligand [46, 47] and has been widely used to study the role of PPAR*γ* activation in cancer cells [48].

## 4. Role of PPAR*γ* in Tumorigenesis of Gastrointestinal Cancers

As discussed earlier, PPAR*γ* is richly expressed in the normal gastrointestinal epithelium. Significantly high level of PPAR*γ* has been observed in the highly differentiated epithelial cells of the proximal colon [11, 12] and small intestine [12, 49]. In small intestine, PPAR*γ* is particularly expressed at the crypt/villus junction where small intestine epithelial cells cease to proliferate and undergo differentiation to mature to functional villus epithelial cells. These observations indicate that PPAR*γ* might play an important role in the regulation of differentiation of gastrointestinal epithelial cells. The fact that PPAR*γ* is expressed at a much higher level in the proximal colon than in distal colon implies that PPAR*γ* may play a complex role in the colon. The mechanisms by which PPAR*γ* regulates the differentiation of human gastrointestinal epithelial cells are not yet clear, but may involve a collaboration of PPAR*γ* with the transcription factor Hic5 as the transactivation of Hic5 by PPAR*γ* was shown to promote differentiation of intestinal epithelial cells during embryonic development [50].

The expression pattern of PPAR*γ* in colon cancer has been previously reported. Loss-of-function mutations to PPAR*γ* have been reported to be associated with increased propensity of human colon cancers [51] although the mutation of PPAR*γ* is very rare [52]. Many in vitro studies have revealed that activation of PPAR*γ* inhibits proliferation and induces apoptosis of some colon cancer cell lines [53–60]. Compatible with these findings, heteroxygous loss of PPAR*γ* increases the susceptibility of colon and stomach into carcinogen-induced colon cancer and gastric cancer, respectively, [61–63]. Biallelic knockout of PPAR*γ* in colonic epithelial cells resulted in increased tumor incidence and tumor size in APC^+/Min^ mice [64], and Pioglitazone suppressed colon tumor growth in APC^+/Min^ mice in a dose-dependent manner [65–67]. Similar results were reported in studies conducted in other animal models. Troglitazone inhibited the formation of preneoplastic colonic aberrant crypt foci (ACF) in rats treated with either AOM or the combination of AOM with dextran suflate (DSS) [68, 69]. Several PPAR*γ* ligands including pioglitazone, rosiglitazone, and RS5444 have been reported to inhibit ACF formation in AOM-mediated colon cancer models [12, 67]. All these studies have suggested that PPAR*γ* functions as a tumor-suppressor gene and it might be involved in cancer suppression under the physiological conditions.

Carcinogenesis usually results from an imbalanced cell proliferation and apoptosis. The inhibitory effect of PPAR*γ* activation on colon cancer could be attributed to several mechanisms. For example, ligands-induced activation of PPAR*γ* was found to inhibit phosphatidylinositol 3-kinase (PI3K)/Akt signaling and retinoblastoma protein (Rb) dephosphorylation [70], induce expression of cyclin D1 [71] and Bcl-xl/Bcl-2 [72], upregulate p21 and p27 [73–75], interact with XIAP [76], upregulate PTEN [77], and enhance the sensitivity of tumor cells to tumor necrosis factor-related apoptosis-inducing ligand- (TRAIL-) induced cell death [78]. Inhibition of hyperlipidemia, a well-established oncogenic factor for colon cancer, has also been proposed as one of the mechanisms responsible for the inhibitory effect of PPAR*γ* ligand on colon cancer [65, 66].

Although induction of apoptosis by PPAR*γ* agonists has been frequently suggested as one of the mechanisms responsible for their anticancer effects, it is less clear and perhaps controversial whether PPAR*γ*-induced apoptosis is actually receptor-mediated, or apoptosis simply occurs as an off target effect of PPAR*γ* agonists.

To complicate the issues even further is the fact that the inhibitory effect of PPAR*γ* activation on colon cancer formation does not have a universal support. For example, two PPAR*γ* agonists, troglitazone and rosigliatzone, have been reported to promote gastrointestinal tumorigenesis in C57BL/6J APC^Min/+^ mice [11, 79], raising the serious concerns about the possibility that individuals who are on TZDs for T2DM might be at risk for colon cancer. These results were not able to be reproduced by other studies [65, 66]. On the contrary, long-term treatment with high concentrations of TZDs was found to increase the frequency of caecal tumors in wild-type C57BL/6J and C57BL/6J APC^+/1638N^/Mlh^+/−^ double mutant mice [80]. Part of the explanation is that in these mouse model studies, the doses of TZDs used were far greater than the doses that can be tolerated in human. Also, to add to the complexity of the role of PPAR*γ* in colon carcinogenesis, it has been reported that many TZDs may activate PPAR*γ* independent of PPAR*γ* receptor [72, 76, 81–84] but may require the presence of APC gene [61].

The contradictory results reported in these studies have clearly reflected the complexity of the role of PPAR*γ* in colon carcinogenesis. Large-scale in vivo studies using not only PPAR*γ* activators or inhibitors but also in knockout mice, especially colon specific knockout of PPAR*γ*, would generate more valuable data.

## 5. Role of PPAR*γ* during Anti-Inflammatory Process

In recent years, the causative link between inflammation and cancer has attracted considerable attention. The mechanism and molecular pathways of chronic inflammation leading to CRC development have been discussed in much detail in a recent review [85]. It has been commonly agreed that in the development of CRC, inflammatory bowel diseases (IBD, which includes ulcerative colitis and Crohn's disease) are among the major risk factors. These risk factors are particularly important in children and young adults of less than 30 years of age [86, 87].

There is an ample amount of evidence suggesting a role of PPAR*γ* in inflammatory processes. PPAR*γ* expression is reduced in ulcerative colitis [88]. PPAR*γ* has been identified as anti-inflammatory molecules in IBD [89]. Mice with a targeted disruption or elimination of the PPAR*γ* gene in intestinal epithelial cells showed an increased susceptibility to dextran sulfate sodium- (DSS-) induced IBD [90].

As macrophages play important role in the anti-inflammatory effect in the colon, its correlation with PPAR*γ* has been actively studied. It has been shown that mice with a targeted disruption of the PPAR*γ* gene in the macrophages of the intestinal epithelia also showed an increased susceptibility to DSS-induced IBD [90, 91], suggesting an important anti-inflammatory role of PPAR*γ*.

However, 5-aminosalicylic acid (5-ASA) is an anti-inflammatory drug widely used in the treatment of IBD. It has been reported that 5-ASA could bind to and activate PPAR*γ* [92]. Binding of 5-ASA to PPAR*γ* receptor was found to be similar to the crystal orientation of the TZD head group of rosiglitazone. These observations indicated that 5-ASA exerted its anti-inflammatory effect through activating PPAR*γ* pathway. Based on these observations, PPAR*γ* ligands have been considered a group of potentially useful therapeutic agents for CRC and IBD [93, 94]. In a clinical trial, rosiglitazone was found to produce clinical and endoscopic remission of patients with ulcerative colitis in the majority of patients although this study was limited by its low number of patients [95].

Nonsteroidal anti-inflammatory drugs (NSAIDs) are also a group of PPAR*γ* activators. These agents are a widely used as anti-inflammatory drugs and pain killers. These agents can inhibit cyclooxygenase (COX) enzymes [96], and have a chemopreventive effect in various cancers, including CRC and gastric cancer [97, 98]. It has been reported that the chemopreventive effect of NSAIDs in gastrointestinal cancers was mechanistically attributable to their ability to activate PPAR*γ*.

It is now widely accepted that activation of PPAR*γ* may be an important mechanism responsible for the anti-inflammatory, and anticancer effects of most, if not all Cox inhibitors. For example, Curcumin, a widely recognized dietary agent with a strong ability to inhibit NF-*κ*B and COX-2, was found to activate the PPAR*γ* pathway in colon cancer [99]. Other COX-2 inhibitors such as indomethacin and sulindac sulphide also were shown to activate PPAR*γ* pathways [100]. In addition, inhibition of Cox-2 and activation of PPAR*γ* may have synergistic effect in inhibiting the growth of certain cancers such as pancreatic cancer [101].

The mechanisms by which COX inhibition leads to PPAR*γ* activation are not clearly defined, but may be possibly due to antagonizing NF-*κ*B activity, suppressing IL-8 and iNOS expressions, or increased production of prostaglandins derivertives such as 15d-PGJ2 [102, 103].

Overall, those data suggested that activation of PPAR*γ* exerts an anticancer effect partially through anti-inflammatory function.

## 6. Role of PPAR*γ* in Epithelial Mesenchymal Transition (EMT) and Tumor Invasion

Metastasis is a subsequent behavior of all malignant tumors, and often the cause of cancer mortality. EMT plays an important role during tumor metastasis. Some of the proteins involved in EMT could be utilized as potential prognostic markers or therapeutic targets.

EMT is regulated by multiple signaling pathways [104]. The process starts from ligand-induced activation of tyrosine kinase receptors. The important ligands that may activate the tyrosine kinase receptors include epidermal growth factor (EGF), fibroblast growth factor (FGF), insulin-like growth factor (ILGF), and hepatocyte growth factor (HGF). Binding of these ligands to receptors alters the functions of some of the down-stream target genes: downregulation of the E-cadherin gene via the transcription factor Snail pathway; or directly affects cell adhesion and/or the cytoskeletal dynamics. Notch, Hedgehog, and NF-*κ*B signaling pathways have also been found to be involved in EMT.

It has been reported that PPAR*γ* promotes EMT by Rho GTPase-dependent activation of ERK1/2 in intestinal epithelial cells [105]. RS5444 (a novel third-generation thiazolidinedione derivative) caused dramatic changes in cellular morphology, which were associated with increased motility and diminished cellular adherence in nontransformed rat intestinal epithelial cells (RIEs). These data suggest novel effects of PPAR*γ* on cell-cell and cell-matrix interactions [49]. However, the precise role and mechanism by which PPAR*γ* regulates EMT and cancer metastasis are not yet well defined.

## 7. Role of PPAR*γ* in Angiogenesis

Angiogenesis plays an important role in the development and metastasis of all solid cancers. The regulatory role of PPAR*γ* in angiogenesis has been demonstrated in vitro and in vivo, as reviewed in details elsewhere [48, 106]. The effect of PPAR*γ* ligands on angiogenesis is bidirectional, possibly depends on cell types and specific pathways involved. Most of the studies showed that PPAR*γ* ligands inhibit angiogenesis, but opposite results have been reported. For example, 15d-PGJ2 inhibits angiogenesis via upregulation of HGF, VEGF, Flt-1 (VEGF receptor-1), and Flk/KDR (VEGF receptor-2). The same agent may also stimulate angiogenesis via the induction of heme oxygenase-1 (HO-1), endothelial nitric-oxide synthase, and hypoxia-inducible factor-1a (HIF-1) [48]. Large-scale studies will have to be conducted to reveal the role of PPAR*γ* ligands in angiogenesis in a particular cancer.

## 8. Current Clinical Trials

Although many in vitro and in vivo data have demonstrated a potential therapeutic role of PPAR*γ* ligands in many cancers, the results from clinical trials are limited and the efficacy of PPAR*γ* ligands in most cancers was less satisfactory. The poor outcome may be partially related to the fact that most of these clinical trails that turned out to be negative on therapeutic effect were conducted in patients with refractory and advanced solid tumors, which are notoriously refractory to most of the available therapeutic approaches.

Table 1 lists some of the clinical trials on treatment of different human cancers by PPAR*γ* ligands. From these data, it is probably more plausible to designate PPAR*γ* ligands as a group of biological modifier in human cancers rather than therapeutic agents.

PPAR*γ* agonists are generally well tolerated. The major adverse effects are gastrointestinal toxicity, include diarrhea, vomiting, and abdominal pain [44, 45]. Certain TZDs, such as troglitazone, has been removed from clinical use because of the severe side effects. Overall, it is still inconclusive to state a definite therapeutic role of PPAR*γ* agonists in gastrointestinal cancers.

## 9. Conclusions

In summary, role of PPAR*γ* in CRC is rather controversial. PPAR*γ* ligands may exert therapeutic effects on colon cancer through a PPAR*γ*-dependent and a PPAR*γ*-independent pathway. The therapeutic effects of the current PPAR*γ* ligands in colon cancer are less optimal. Thus, new PPAR*γ* ligands are currently under development. Exploration of combinational therapy using PPAR*γ* ligands and other therapeutic drugs should be encouraged.

## Figures and Tables

**Table 1:** Some of the clinical trials on the role of PPAR**γ** ligands on CRC.

Authors	Drug used	Number of patients	Combined treatment	Tumors	Effect
Tepmongkol et al.	Rosiglitazone	23	Radioiodine	Thyroid carcinoma	Responded
Hau et al.	Pioglitazone	14	Capecitabine/Temozolomide and Rofecoxib	Glioma	Partial response
Kebebew et al.	Rosiglitazone	10	Radioiodine	Thyroid carcinoma	Responded
Schwartz et al.	LY293111	38	No	Solid tumors	No response
Baetz et al.	Ly293111	28	Irinotecan	Solid tumors	No response
Demetri et al.	Troglitazone	3	No	Liposarcoma	Responded
Smith et al.	Rosiglitazone	106	No	Prostate carcinoma	No response
Tenenbaum et al.	Bezafibrate	3011	No	Colon cancer	Responded
Read et al.	Rosiglitazone	23	Bexarotene	Solid tumor	No response
Debrock et al.	Rosiglitazone	12	Pretreatment	Liposarcoma	negative
Burstein et al.	Troglitazone	22	Prechemotherapy or Prehormonal	Breast cancer	No response
Kulke et al.	Troglitazone	25	No	Colon cancer	No response
Mueller et al.	Troglitazone	41	Preandrogen deprivation	Prostate cancer	No response
